# The H3K79me3 methyl-transferase Grappa is involved in the establishment and thermal plasticity of abdominal pigmentation in *Drosophila melanogaster* females

**DOI:** 10.1038/s41598-024-60184-6

**Published:** 2024-04-25

**Authors:** Raphaël Narbey, Emmanuèle Mouchel-Vielh, Jean-Michel Gibert

**Affiliations:** Laboratoire de Biologie du Développement, UMR 7622, CNRS, Institut de Biologie Paris-Seine (IBPS), Sorbonne Université, 9 Quai St-Bernard, 75005 Paris, France

**Keywords:** Developmental biology, Genetics

## Abstract

Temperature sensitivity of abdominal pigmentation in *Drosophila melanogaster* females allows to investigate the mechanisms underlying phenotypic plasticity. Thermal plasticity of pigmentation is due to modulation of *tan* and *yellow* expression, encoding pigmentation enzymes. Furthermore, modulation of *tan* expression by temperature is correlated to the variation of the active histone mark H3K4me3 on its promoter. Here, we test the role of the DotCom complex, which methylates H3K79, another active mark, in establishment and plasticity of pigmentation. We show that several components of the DotCom complex are involved in the establishment of abdominal pigmentation. In particular, Grappa, the catalytic unit of this complex, plays opposite roles on pigmentation at distinct developmental stages. Indeed, its down-regulation from larval L2 to L3 stages increases female adult pigmentation, whereas its down-regulation during the second half of the pupal stage decreases adult pigmentation. These opposite effects are correlated to the regulation of distinct pigmentation genes by Grappa: *yellow* repression for the early role and *tan* activation for the late one. Lastly, reaction norms measuring pigmentation along temperature in mutants for subunits of the DotCom complex reveal that this complex is not only involved in the establishment of female abdominal pigmentation but also in its plasticity.

## Introduction

Phenotypic plasticity is the ability of a given genotype to produce different phenotypes in response to distinct environmental conditions^1^. It is widely observed in the wild and is often an adaptation to predictable environmental fluctuations such as seasonal variations. Furthermore, phenotypic plasticity is thought to facilitate evolution, as a phenotype initially induced by the environment can become subsequently fixed by genetic assimilation. A few empirical studies give support to such models of evolution called “Flexible stem hypothesis” or “Plasticity first evolution”^2–7^. Despite these important roles in adaptation and evolution, the molecular mechanisms underlying phenotypic plasticity are only beginning to be unraveled. Here, we use the abdominal pigmentation of *Drosophila melanogaster* females as a model to study the mechanisms involved in phenotypic plasticity. Abdominal pigmentation is sensitive to temperature, as females grown at 18 °C are darker than those grown at 29°C^8^. Indeed, the expression of several pigmentation enzyme coding genes (called hereafter pigmentation genes), such as *tan* and *yellow,* is regulated by temperature^9,10^. In particular, *tan*, encoding an enzyme involved in production of black and brown melanin^11^, is expressed 7 times more at 18 °C than 29 °C in young females^9^. Environmental conditions can affect gene expression through modifications of chromatin structure, mainly via histone modifications, nucleosome remodeling or DNA methylation^12–14^. We have previously shown that the H3K4 methyl-transferase Trithorax, that deposits the H3K4me3 active histone mark, is involved in the establishment and plasticity of Drosophila pigmentation by activating *tan* expression through H3K4 methylation of its promoter^9^. Furthermore, the quantity of H3K4me3 on the *tan* promoter is modulated by temperature. Here, we investigate the role of the DotCom complex, involved in the deposition of the active histone mark H3K79me3, on pigmentation gene expression and thermal plasticity of abdominal pigmentation.

The mono-, di- and trimethylation of H3K79 is catalyzed by Dot1 (Disruptor of telomeric silencing-1), a non-SET domain-containing histone lysine methyl-transferase first identified in yeast^15^. This enzyme, encoded by *grappa* (*gpp*) in *Drosophila*^16^, is highly conserved from yeast to vertebrates and seems to have a dual role. In yeast and *Drosophila*, telomeric silencing is disrupted in both *Dot1/gpp* loss- and gain- of function mutants^16–18^. Moreover, in *Drosophila*, *gpp* mutants show phenotypes indicative of disruption of both Polycomb*-*Group silencing complexes (PcG) and Trithorax-Group activating complexes (TrxG)^16^. In mammals, Dot1 is involved in embryonic development and heterochromatin organization^19^. Altogether, these data reveal an important role of H3K79 methylation in the regulation of developmental genes.

Genome-wide profiling studies in different mammalian cell lines show that H3K79me2 and H3K79me3 marks localize to the promoter-proximal regions of actively transcribed genes, with a good correlation with high transcriptional levels^20^. In yeast, nearly 90% of the genome is methylated on H3K79 residues^18^. In human, these methylations are associated with activation at single-copy genes^21^. H3K79me2 is enriched in euchromatin whereas H3K79me3 is enriched in repeated sequences and chromocenter^22^. In *Drosophila*, the level of H3K79me3 is positively correlated with gene activity^23^, which is consistent with some of the phenotypes previously reported for *gpp* mutants such as anterior transformations of the posterior abdominal segments^16^.

Interestingly, other histone modifications facilitate H3K79 methylation by Dot1. In yeast, the mono-ubiquitination of histone H2B on lysine 123 (H2BK123ub) is required for an efficient deposition of methylations on H3K79 (H3K79me2/3) by Dot1^24,25^. Structural studies showed that the interaction between Dot1 and H2BK123ub, by reducing the distance between Dot1 and the nucleosome, allows a higher methylation efficiency^26^. Furthermore, the acetylation of histone H4 on lysine 16 (H4K16ac) is also involved in the regulation of Dot1 activity by structuring and stabilizing the Dot1-nucleosome interaction^27^.

The Dot1/Gpp enzyme belongs to a large multi-subunits complex called DotCom. In mammals, this complex is composed of AF10, AF17, AF9, ENL, Skp1 and TRRAP. In *Drosophila*, the complex comprises the corresponding homologues: Alhambra (Alh, homologue of both AF10 and AF17), EAR (homologue of AF9 and ENL), NippedA (homologue of TRRAP) and SkpB (homologue of Skp1)^28^. We show here that components of the *Drosophila* complex DotCom (Alhambra, Ear and Gpp) are involved in the establishment and thermal plasticity of abdominal pigmentation.

## Results

### The DotCom complex is involved in pigmentation establishment

**Figure 1:**
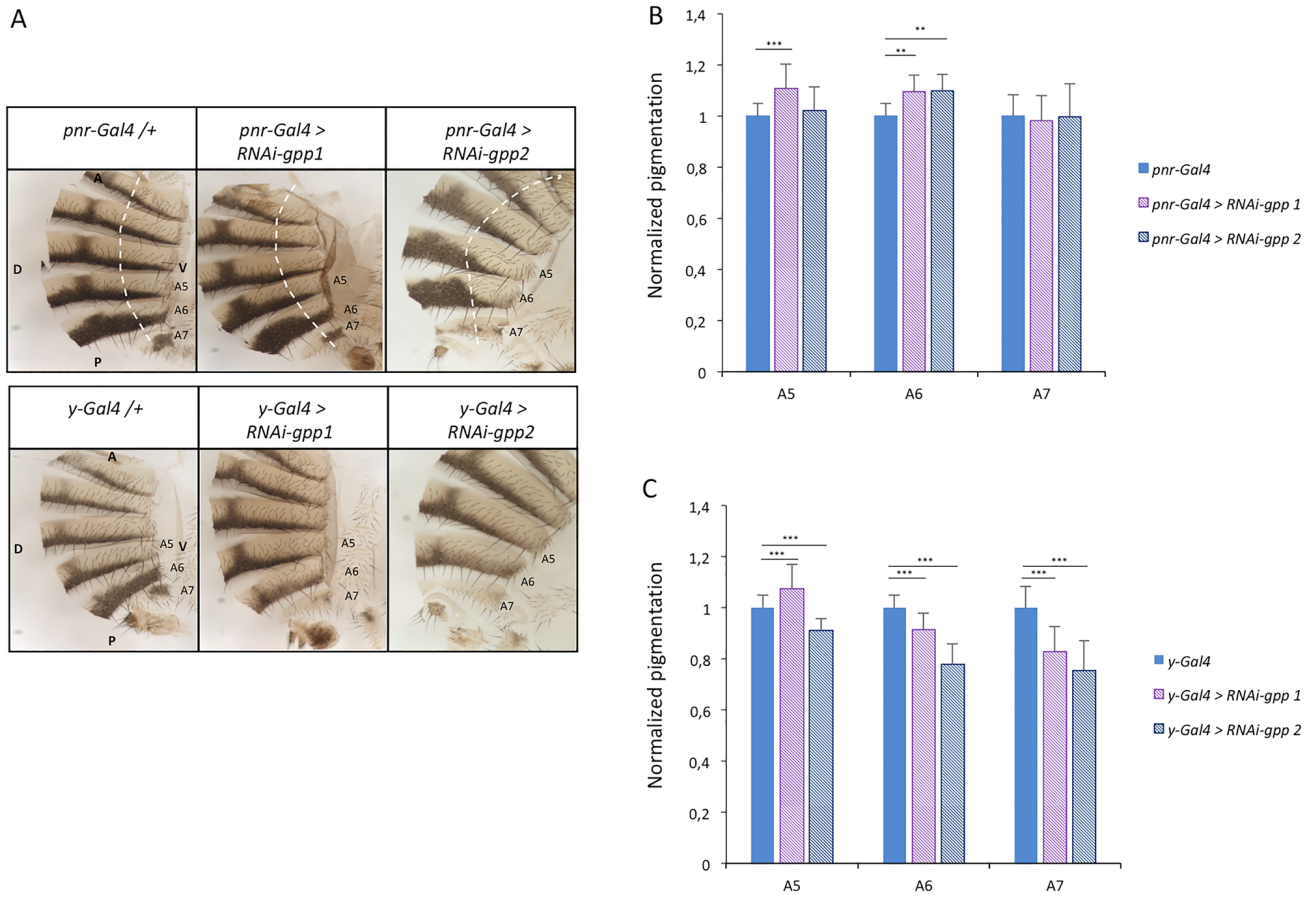
The methyl-transferase Gpp is involved in the establishment of pigmentation. (**A**) Abdominal cuticles of females either control (*pnr-Gal4/* + *or y-Gal4/* +) or RNAi against *gpp* (*UAS-RNAi-gpp1* and *UAS-RNAi-gpp2*) driven by *pnr-Gal4 and y-Gal4*. Cuticles were cut just beyond the dorsal midline. Hemi-abdomens are shown. Dashed white lines mark the right borders of the *pnr* driver expression domain. Abdominal tergites A5 to A7 are indicated. A: anterior; P: posterior; V: ventral; D: dorsal. B, C) Quantification of A5, A6 and A7 pigmentation of *UAS-RNAi-gpp1* and *UAS-RNAi-gpp2* females driven by *pnr-Gal4* (**B**) or *y-Gal4* (**C**), compared to *pnr-Gal4/* + and *y-Gal4/* + controls (30 females *per* genotype, error bars correspond to standard deviations, Tukey post-hoc test following ANOVA on aligned rank transformed data; ***: *p* < 0.001, **: *p* < 0.01).

In order to test the role of the DotCom complex on the establishment of pigmentation in *Drosophila* females, we first down-regulated *gpp*, encoding the histone methyl-transferase, by using two *UAS-RNAi* transgenes (*RNAi-gpp1* and *RNAi-gpp2*) and two drivers, *pnr-Gal4* and *y-Gal4. pnr-Gal4* is an « early » driver, as *pnr* encodes a transcription factor expressed from the embryo to the adult, along the dorso-longitudinal line^29^ (Supplementary Fig. 1). *y-Gal4* is a « late » driver, as *yellow* (*y*) encodes a pigmentation gene expressed in the abdominal epidermis during the second half of the pupal stage^30^ (Supplementary Fig. 1). We observed a significant effect of both *RNAi* transgenes on pigmentation but, surprisingly, these effects were opposite depending on the driver (Fig. 1, Supplementary table 1, Supplementary file 1). *gpp* down-regulation with the *pnr-Gal4* driver induced an increase in pigmentation in A5 and A6 abdominal segments, whereas with the *y-Gal4* driver pigmentation decreased in A6 and A7. As the main difference between the two drivers is their stage of expression, this result suggests that Gpp plays different roles on pigmentation depending on the developmental stage. The *UAS-RNAi-gpp1* line (hereafter called *UAS-RNAi-gpp)* was used for the following experiments, since the two *gpp RNAi* transgenes gave very close results.

**Figure 2:**
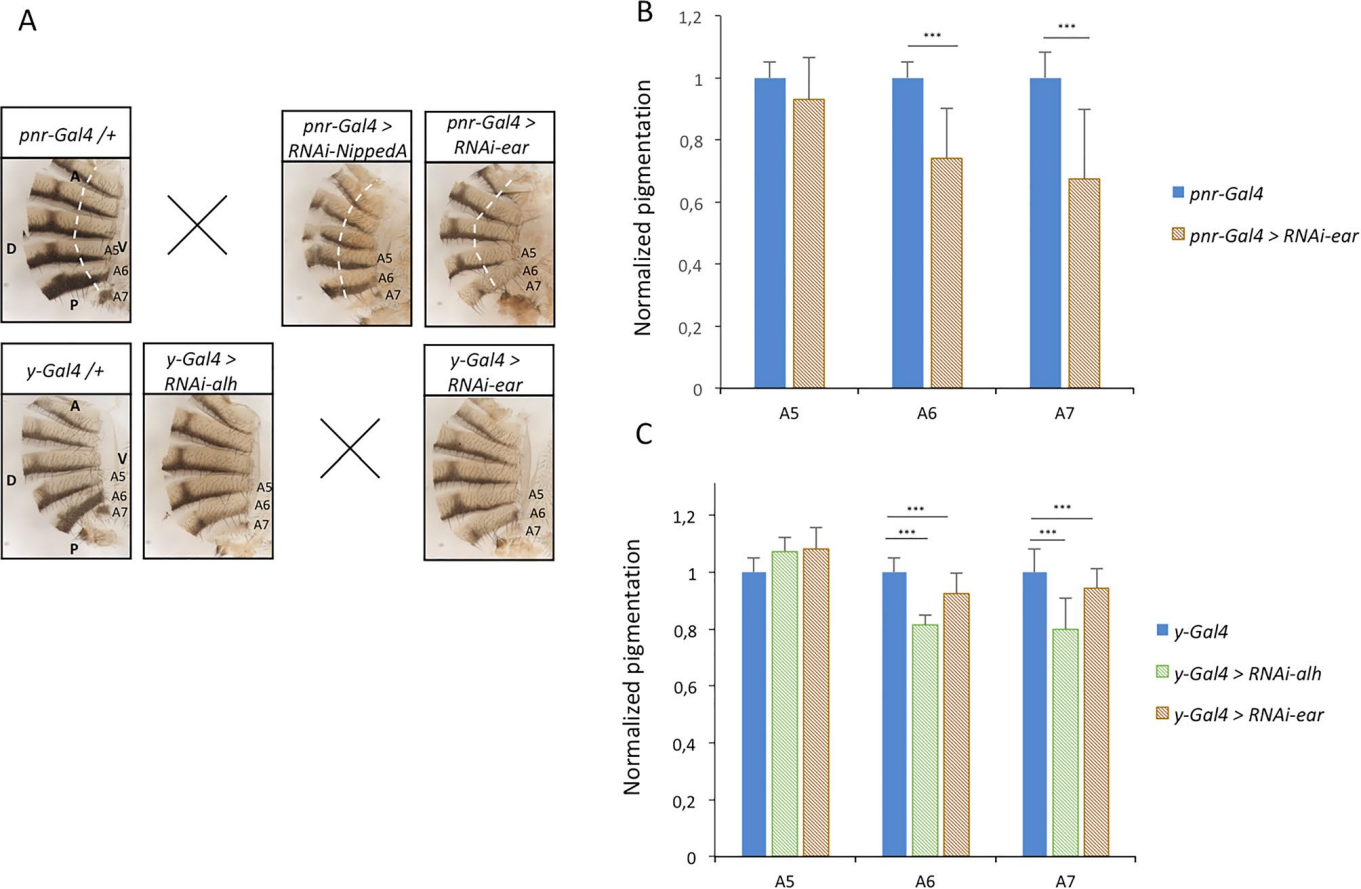
Subunits of the DotCom complex are involved in the establishment of pigmentation. (**A**) Abdominal cuticles of control females (*pnr-Gal4/* + or *y-Gal4/* +) and females in which genes encoding subunits of the DotCom complex have been inactivated using *RNAi* transgenes driven by *pnr-Gal4* and *y-Gal4*. Cuticles were cut just beyond the dorsal midline. Hemi-abdomens are shown. Dashed white lines mark the right borders of *pnr* driver expression domain. Abdominal tergites A5 to A7 are indicated. A: anterior; P: posterior; V: ventral; D: dorsal. The crosses indicate lethality. (**B**,**C**) Quantification of A5, A6 and A7 pigmentation of *RNAi* females driven either by *pnr-Gal4* (**B**) or *y-Gal4* (**C**), compared to *pnr-Gal4/* + and *y-Gal4/* + controls (n = 30 *per* genotype, error bars correspond to standard deviations, t-tests; ***: *p* < 0.001).

To further confirm the role of the DotCom complex on the establishment of pigmentation, *UAS-RNAi* transgenes against the three other subunits of the complex were used (Fig. 2, Supplementary table 1). Using the *pnr-Gal4* driver, only *ear* down-regulation gave interpretable results and induced a decrease of pigmentation. For *NippedA*, the flies presented cuticles incorrectly fused along the dorsal midline that precluded the analysis of pigmentation, whereas for *alh* no larvae hatched. With the *y-Gal4* driver, *ear* and *alh* down-regulation induced a decrease of pigmentation, similar to the effect of *gpp* down-regulation. *NippedA* down-regulation was lethal after the second larval stage.

In conclusion, these results showed that the DotCom complex and notably the Grappa sub-unit that encodes a H3K79 histone methyl-transferase are involved in the establishment of abdominal pigmentation but also in some essential developmental mechanisms.

### Gpp plays opposite roles on pigmentation depending on the developmental stage

**Figure 3:**
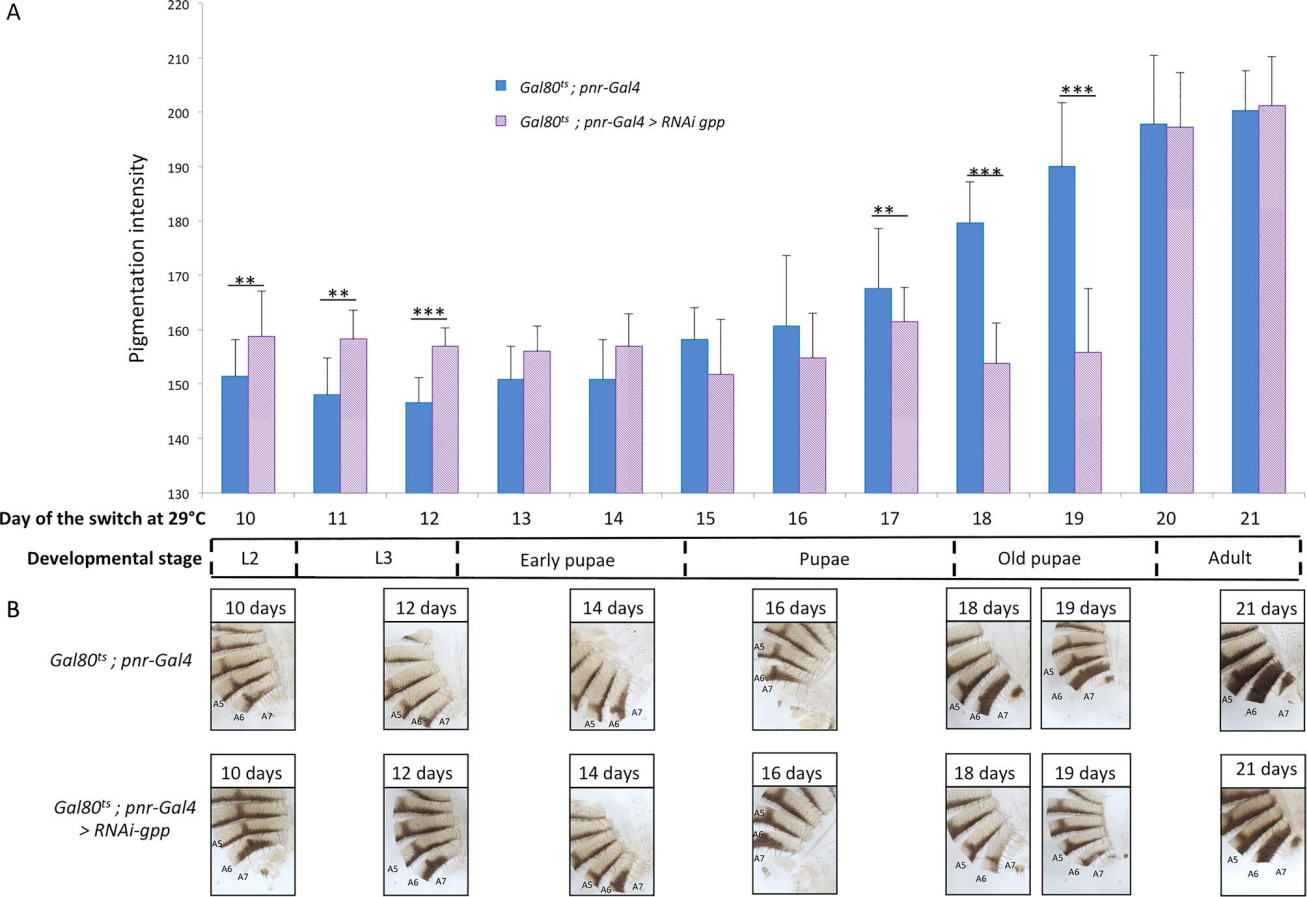
Gpp plays opposite roles on pigmentation depending on the developmental stage. (**A**) Quantification of A6 pigmentation in *UAS-RNAi-gpp* flies driven by *Gal80*^*ts*^*; pnr-Gal4.* The RNAi transgene was activated by shifting a pool of developing flies from 18 to 29 °C each day, from day 10 (L2 stage) to day 21 (adult stage). n = 30 *per* condition, error bars correspond to standard deviations, t-test; ***: *p* < 0.001. (**B**) Abdominal cuticles of *Gal80*^*ts*^; *pnr-Gal4* control and *RNAi-gpp* females with the corresponding day of activation of the RNAi transgene.

The major difference between the two drivers used to down-regulate *gpp* is the developmental stage at which they are expressed. Therefore, the opposite effects of *gpp* down-regulation on pigmentation revealed by these drivers could reflect the distinct roles played by *gpp* at different developmental stages. To test this hypothesis, we used a temperature-sensitive Gal80 repressor, Gal80^ts^, in combination with the *pnr-Gal4* driver. This system allowed to repress *gpp* at different developmental stages. The *UAS-RNAi-gpp* was activated by shifting tubes containing developing flies from 18 to 29 °C between day 10 of development (L2 stage) and day 21 (young adult stage), and quantifying the A6 segment pigmentation in adult females. As temperature itself affects pigmentation, control flies were treated identically. Between days 10 and 12, *gpp* down-regulation induced an increase of pigmentation in A6, similarly to the phenotype observed with *pnr-Gal4*. By contrast, between days 17 and 19, a decrease of pigmentation was observed, similar to the phenotype induced by *y-Gal4* (Fig. 3, Supplementary table 2). These results show that *gpp* is involved in different ways in pigmentation establishment during development, regulating the gene network involved in melanin production negatively during larval development and positively during the second half of the pupal life.

### Gpp regulates different pigmentation genes depending on the developmental stage

**Figure 4:**
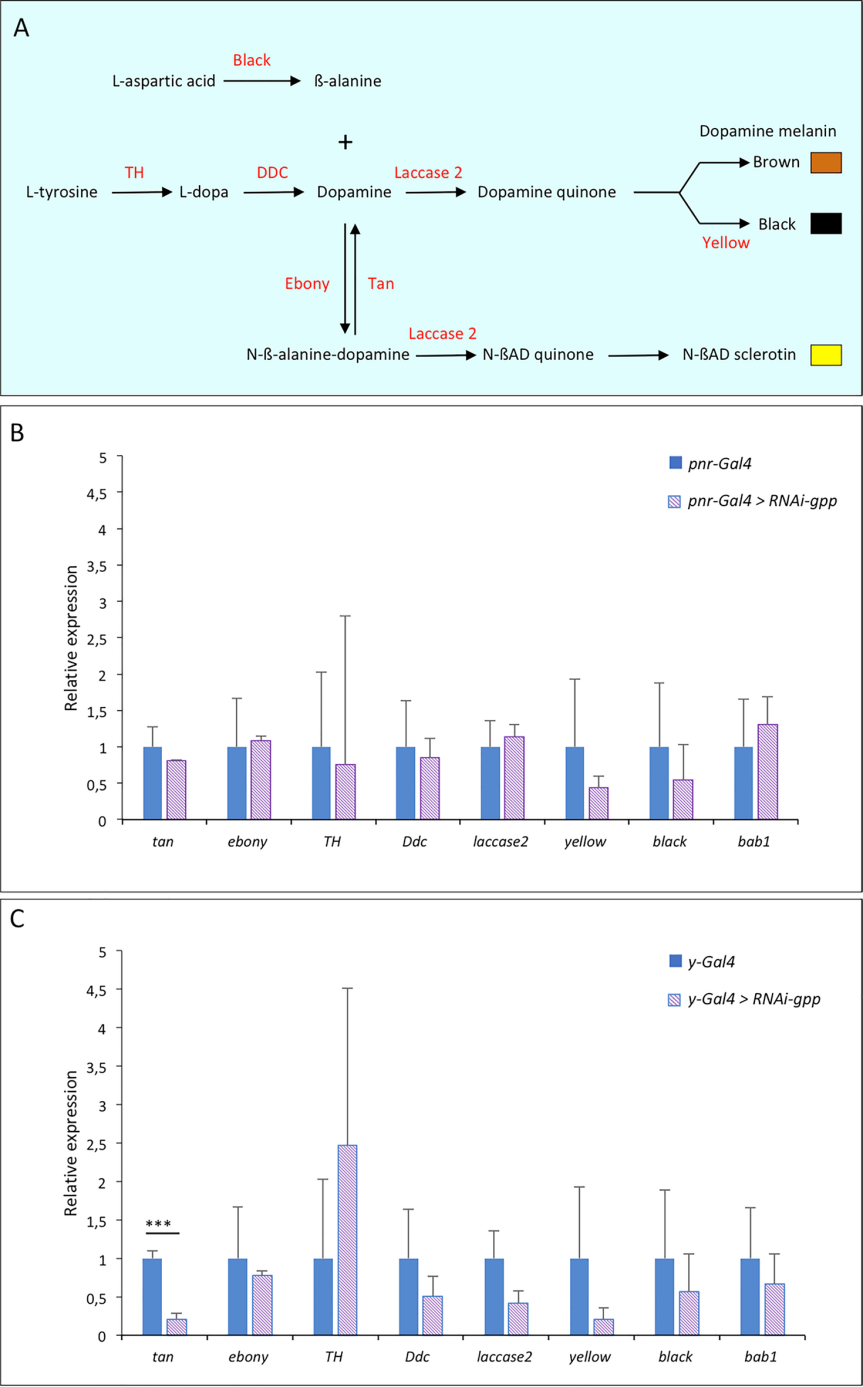
Gpp activates the expression of the pigmentation gene *tan*. (**A**) Synthesis pathway of cuticular pigments^9^. The enzymes are highlighted in red. (**B**,**C**) RT-qPCR quantification of pigmentation genes, encoding these enzymes, and *bab1* expression in posterior abdomen epidermes (A5, A6 and A7 segments) of young *RNAi-gpp* females driven by *pnr-Gal4* (**B**) or *y-Gal4* (**C**) (n = 3, error bars correspond to standard deviations, t-test; ***: *p* < 0.001). The geometric mean of *RP49* and *Spt6* reference gene expression was used for normalization.

**Figure 5:**
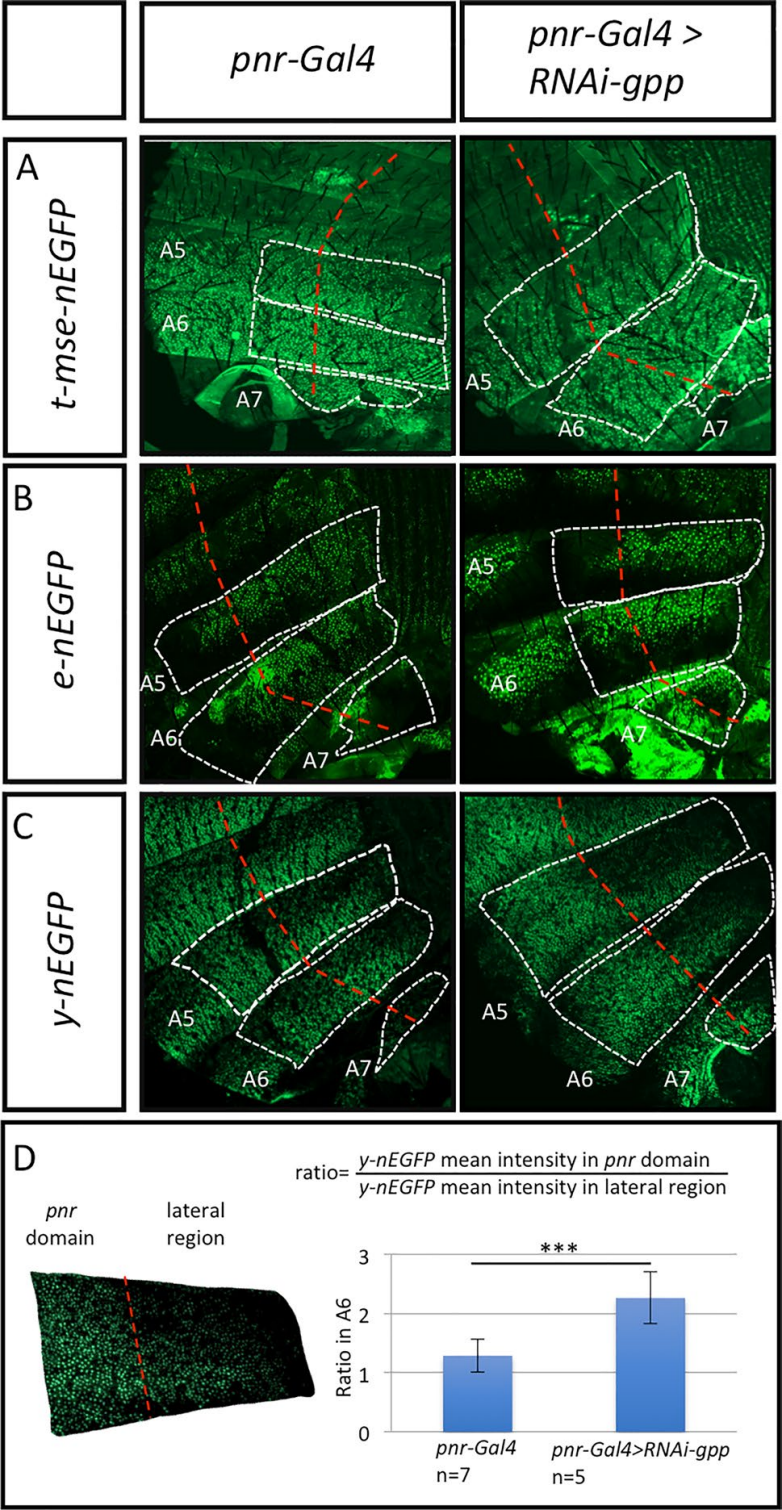
Gpp represses the expression of the pigmentation gene *yellow*. In (**A**–**C**), the dashed white lines represent the right hemi-segments. The dashed red line marks the limit between the dorsal *pnr-Gal4* expression domain (to the left of the line) and the lateral region (to the right of the line) used as an internal control. (**A**,**B**) Effect of *gpp* down-regulation on *tan* (**A**) and *ebony* (**B**) nEGFP reporters. Compared to the control *pnr-Gal4* (left), *gpp* down-regulation (right) has no effect on the expression of nEGFP driven neither by *tan* nor by *ebony*. (**C**) Effect of *gpp* down-regulation on the *yellow* nEGFP reporter transgene*.* Compared to the control *pnr-Gal4* (left), *gpp* down-regulation (right) increases the expression of nEGFP, which extends more anteriorly in the *pnr-Gal4* expression domain. (**D**) Computation of the ratio of the mean intensity of *yellow* nEGFP in the *pnr* domain over that in the lateral region (used as internal control) for *pnr-Gal4* and *pnr-Gal4* > *RNAi-gpp*. Error bars correspond to standard deviations. t-test; ****: *p* < 0.001.

As Gpp regulates melanin production, we aimed to identify the pigmentation genes targeted, directly or indirectly, by Gpp. The pigmentation genes encode the enzymes involved in the cuticular pigment synthesis pathway, which is very dynamic. These enzymes act sequentially to produce the different pigments of the cuticle (brown and black melanin and yellow NßAD sclerotin) (Fig. 4A)^9^. Modification in melanin production could result from a change in expression of genes acting early in the pathway, such as *TH* or *DDC*. Alternatively, it could be caused by a deregulation of genes acting downstream, involved in the balance between yellow pigments and melanins, such as *tan*, *ebony* and *yellow*. We thus performed RT-qPCR experiments on A5, A6 and A7 epidermes of young *RNAi-gpp* females driven by *y-Gal4* or *pnr-Gal4* to measure the expression of the genes encoding the enzymes from the pigment synthesis pathway (Fig. 4B,C, Supplementary File 2, Supplementary Table 3). With the *pnr-Gal4* driver, no significant difference in pigmentation gene expression was observed (Fig. 4B). This could be due to the fact that, *pnr* being expressed only around the dorso-longitudinal line, the tissue expressing *pnr* was too diluted in the total abdominal epidermis. To bypass this issue, we observed the expression of nEGFP transgenes reporting the expression of the pigmentation genes *yellow*^31^*, ebony*^32^ and *tan*^33^ in *UAS-RNAi-gpp* flies driven by *pnr-Gal4* (Fig. 5). No change of GFP expression was observed for *tan* and *ebony* reporters (Fig. 5A,B). In contrast, for the *yellow* reporter, GFP expression increased in the expression domain of *pnr* compared to controls (Fig. 5C). The ratio of GFP intensity in the *pnr* domain to that in the lateral region (used as an internal control) was 1.76 times higher in *pnr-Gal4* > *RNAi gpp* flies than in *pnr-Gal4* control flies (Fig. 5D; Supplementary Table 4). *yellow* being involved in production of black melanin^34^, this observation is consistent with the pigmentation phenotype of *pnr-Gal4* > *RNAi-gpp* flies. Therefore, Gpp represses *yellow* expression.

With the *y-Gal4* driver, the expression of *tan*, involved in production of brown and dark melanin, decreased, which is consistent with the pigmentation phenotype of *y-Gal4* > *RNAi-gpp* flies (Fig. 4C). Therefore, Gpp activates *tan* expression. We have shown previously that the Bric-à-Brac transcription factors (Bab1 and Bab2) participate in the establishment and plasticity of pigmentation by repressing *tan*^35^. In order to test whether Gpp could activate *tan* through *bab* regulation, we quantified *bab1* expression (Fig. 4B,C). No significant change in *bab1* expression was observed, suggesting that Gpp regulates *tan* independently of Bab.

### The DotCom complex is involved in thermal plasticity of abdominal pigmentation

**Figure 6:**
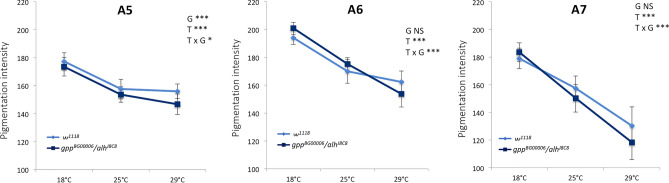
The DotCom complex plays a role in the thermal plasticity of abdominal pigmentation. Quantification of A5, A6 and A7 pigmentation of *gpp*^*BG00006*^*/alh*^*J8C*^mutant and control females raised at 18 °C, 25 °C or 29 °C (n = 30 *per* condition, error bars correspond to standard deviations). G: effect of genotype; T: effect of temperature; T x G: effect of the interaction between genotype and temperature. ANOVA on aligned rank transformed data was performed for A5. ANOVA were performed for A6 and A7 segments. * *p* < 0.05; ***: *p* < 0.001. NS: non significant.

The DotCom complex being involved in pigmentation establishment, we then wondered whether it also participates in the regulatory network mediating thermal plasticity of abdominal pigmentation. To answer to this question, as thermal sensitivity of the UAS/Gal4 system prevented the use of RNAi lines, we studied a double heterozygous loss-of-function mutant for *gpp* (*gpp*^*BG00006*^) and *alh* (*alh*^*J8C8*^) to strongly destabilize the DotCom complex. Flies were grown at 18 °C, 25 °C and 29 °C. Their pigmentation was quantified in A5, A6 and A7 and compared to those of control flies (Fig. 6, Supplementary Table 5, Supplementary File 3). Pigmentation of both mutant and control females were sensitive to temperature (“T effect”). The mutant females were significantly lighter than the control females (“G effect”) for A5 but not for A6 and A7. A very strong interaction, visible as the curves of the two genotypes crossed each other, was observed between genotype and temperature (“T x G effect”) for A6 and A7 and to a lesser extent for A5. This significant interaction between genotype and temperature revealed that thermal plasticity was modified in *gpp*^*BG0000*^*/alh*^*J8C8*^ females as compared to control females. These results show that the DotCom complex participates in thermal plasticity of abdominal pigmentation.

## Discussion

We show here that the DotCom complex is involved in the establishment and thermal plasticity of the abdominal pigmentation of *Drosophila melanogaster* females. Interestingly, Gpp, the histone methyl-transferase of the complex, participates in opposite ways in the establishment of pigmentation depending on the developmental stage. Indeed, when *gpp* is down-regulated from the L2 to the L3 stages, melanin production is higher, whereas when it is down-regulated later, during the second half of the pupal life, melanin production is lower. These two effects on pigmentation are mediated by the regulation of two pigmentation genes involved in melanin production, *i.e. yellow* for the early effect and *tan* for the late effect. Thus, Gpp regulates different targets during the establishment of female *D. melanogaster* abdominal pigmentation. It is likely that *yellow* is not a direct target of Gpp. Indeed, *yellow* starts to be expressed in the late pupal stage. However, overexpression of the *yellow* GFP reporter is observed when *gpp RNAi* is induced with the early driver *pnr-Gal4, i.e.* prior to expression of the endogenous gene. In addition, temperature shift experiments showed that the effect on pigmentation of *gpp* down-regulation driven by *pnr-gal4* is determined during the larval life. Concerning *tan*, we observed no significant reduction of the H3K79me3 level on its promoter, second exon and enhancer in *y-Gal4* > *RNAi-gpp* females (Supplementary Fig. 2, Supplementary File 4, Supplementary table 3), suggesting that, like *yellow*, *tan* is not directly regulated by Gpp. In contrast, we have shown previously that the histone methyl-transferase Trx participates in the deposition of the H3K4me3 active mark on *tan*^9^. The opposite roles of Gpp through the regulation of distinct pigmentation genes could explain its involvement in the thermal plasticity of abdominal pigmentation. Indeed, the expression of *yellow* and *tan* is modulated by temperature^9,10^. Temperature might therefore modulate the balance between the repression of *yellow* and the activation of *tan* by Grappa. This is reminiscent of Abdominal-B who plays also opposite roles on cuticle pigmentation depending on the temperature^36^.

In conclusion, our results clearly show that the DotCom complex and its histone methyl-transferase Gpp are involved in the establishment and the plasticity of abdominal pigmentation, through the regulation of *yellow* and *tan* pigmentation genes. However, the direct targets of this complex mediating this effect in the abdominal epidermis remain unknown. They could be identified by performing ChIP-seq experiments to study the level of H3K79 methylation in the abdominal epidermis of control and *gpp* RNAi flies genome-wide. Moreover, we cannot formally exclude the possibility that the DotCom complex acts independently from its histone methyl-transferase activity in this process. To address this question, we could study mutant flies that lack the catalytic activity of Gpp.

In addition, mechanistic insights could be gained by the study of Bre1 and MOF, depositing H2BK123ub and H4K16ac respectively, that facilitate H3K79 methylation by Gpp. Furthermore, a H3K79 demethylase, KDM2B, has recently been characterized in human cells^37^. It would be interesting to identify the Drosophila homologue of KDM2B and test its role on the establishment and plasticity of abdominal pigmentation.

## Material and methods

### Fly stocks

A *w*^*1118*^ inbred line was used as control. The *pnr-Gal4* (BL-3039)^29^, *y-Gal4* (BL-44267), *UAS-RNAi-alh* (BL-39057)*, UAS-RNAi-gpp1* (BL-42919)*, UAS-RNAi-gpp2* (BL-34842)*, UAS-RNAi-ear* (BL-28068)*, UAS-RNAi-NippedA* (BL-34849)*, alh*^*J8C8*^ (BL-12118) and *gpp*^*BG00006*^ (BL-12802) lines were from the Bloomington Stock Center. In order to be tested in the same genetic background than the *w*^*1118*^ control, *alh*^*J8C8*^ and *gpp*^*BG00006*^ alleles were introgressed in *w*^*1118*^ for 10 generations by following eye colour, as these two alleles are marked by *mini-white*. The *Gal80*^*ts*^*-Gal4* transgene was previously described^9^. The efficiency of the *UAS-RNAi-gpp1* transgene was validated by RT-qPCR (Supplementary Fig. 3, Supplementary File 5, Supplementary table 3). The *t_MSE-nEGFP*, *ebony-nEGFP (ebony ABC* + *intron)* and *yellow-nEGFP (yellow-wing-body-nEGFP)* reporter transgenes were previously described^31–33^.

### Quantification of gene expression by RT-qPCR

Total RNA were extracted from pools of 30 dissected posterior abdominal epidermes of females (A5, A6 and A7) with the RNAeasy Mini kit (Qiagen). Three replicates were performed in all experiments. cDNAs were synthesized with the LunaScript RT SuperMix kit (New England Biolabs) using random primers. RT-qPCR experiments were performed in a CFX96 system using SsoFast EvaGreen SuperMix (Biorad). Expression was quantified following the Pfaffl method^38^ using the geometric mean of the expression of *RP49* and *Spt6* reference genes for normalization^39^. All primers are listed in Supplementary table 3.

### Cuticle and epidermis preparations

5 days old adult females were stored in ethanol 70% during ten days. Abdominal cuticles were cut just beyond the dorsal midline and dehydrated in ethanol 100% during 5 min. After dehydration, cuticles were mounted in Euparal (Roth). For nEGFP observation, pupal and adult abdomens were dissected in PBS, fixed 20 min in 3.7% paraformaldehyde in PBS, washed three times 10 min in PBS, and mounted in Mowiol®.

### Image acquisition and GFP quantification

Cuticles were imaged as described in^35^. Abdominal epidermes of females expressing nEGFP under the control of *t_MSE*, *yellow* and *ebony* regulatory sequences were imaged using a macro-apotome (Zeiss). Young adult females were imaged for *t_MSE-nEGFP* and *ebony-nEGFP* and female pupae for *yellow-nEGFP* as these genes are not expressed at the same developmental stage. The mean intensity of *yellow*-nEGFP was measured using ImageJ. Larvae showing H2B-GFP under the control of *pnr-Gal4* and *yellow-Gal4* were imaged with a binocular equipped with a Leica DC480 digital camera using the Leica IM50 Image Manager software. Pharates showing H2B-GFP under the control of *pnr-Gal4* and *yellow-Gal4* were imaged using an Olympus BX41 fluorescence microscope (objective 4 X) equipped with a Yokogawa spinning disc and a CoolSnapHQ2 camera controlled by Metaview software (Universal Imaging).

### Chromatin immunoprecipitation experiments

Chromatin immunoprecipitation (ChIP) experiments were performed as previously described^9^, with 3 µg of each antibody. Rabbit IgGs (Diagenode) as negative control and anti-H3K79me3 (C15410068, Diagenode) were used. Three replicates of 50 abdominal epidermes (A5, A6 and A7) of young females were analyzed. qPCR were performed on a CFX96 system using the Luna Universal qPCR Master Mix (BioLabs). Primers are listed in Supplementary table 3. Data were normalized against input chromatin.

### Statistical analyses

In Figs. 2, 3, 4 and 5, pigmentation intensity, gene expression quantifications and GFP intensity ratios were compared by t-tests using the appropriate parameters depending on homogeneity of variances as assessed with Levene tests. In Fig. 1, as two RNAi lines fo *gpp* were used, we performed ANOVAs on aligned rank transformed data followed by Tukey post-hoc tests (Supplementary File 1). In Fig. 6, to analyse the effect of *alh*/*gpp* double mutants on pigmentation plasticity, a two-way ANOVA with genotype and temperature as factors was used. Variance homogeneity was checked using Levene tests and normality of residuals using Shapiro–Wilk tests. When variances were not homogeneous or residues not normally distributed, a non-parametric ANOVA on aligned rank transformed data was performed. All ANOVA tests were performed using RStudio (Supplementary File 1; Supplementary File 3).

### Supplementary Information

Supplementary Information 1.

Supplementary Information 2.

Supplementary Information 3.

Supplementary Information 4.

Supplementary Information 5.

Supplementary Information 6.

Supplementary Information 7.

Supplementary Information 8.

Supplementary Information 9.

Supplementary Information 10.

Supplementary Information 11.

Supplementary Information 12.

Supplementary Information 13.

## Data Availability

All data generated or analysed during this study are included in this published article [and its supplementary information files].
