# Supplementary material for: The H3K79me3 methyl-transferase Grappa is involved in the establishment and thermal plasticity of abdominal pigmentation in Drosophila melanogaster females
Source: Sci Rep. 2024 Apr 25;14:9547. doi: 10.1038/s41598-024-60184-6 (PMC11045721; doi:10.1038/s41598-024-60184-6)
Supplement: Supplementary file 1 — Supplementary Information 1. [file 41598_2024_60184_MOESM1_ESM.docx]

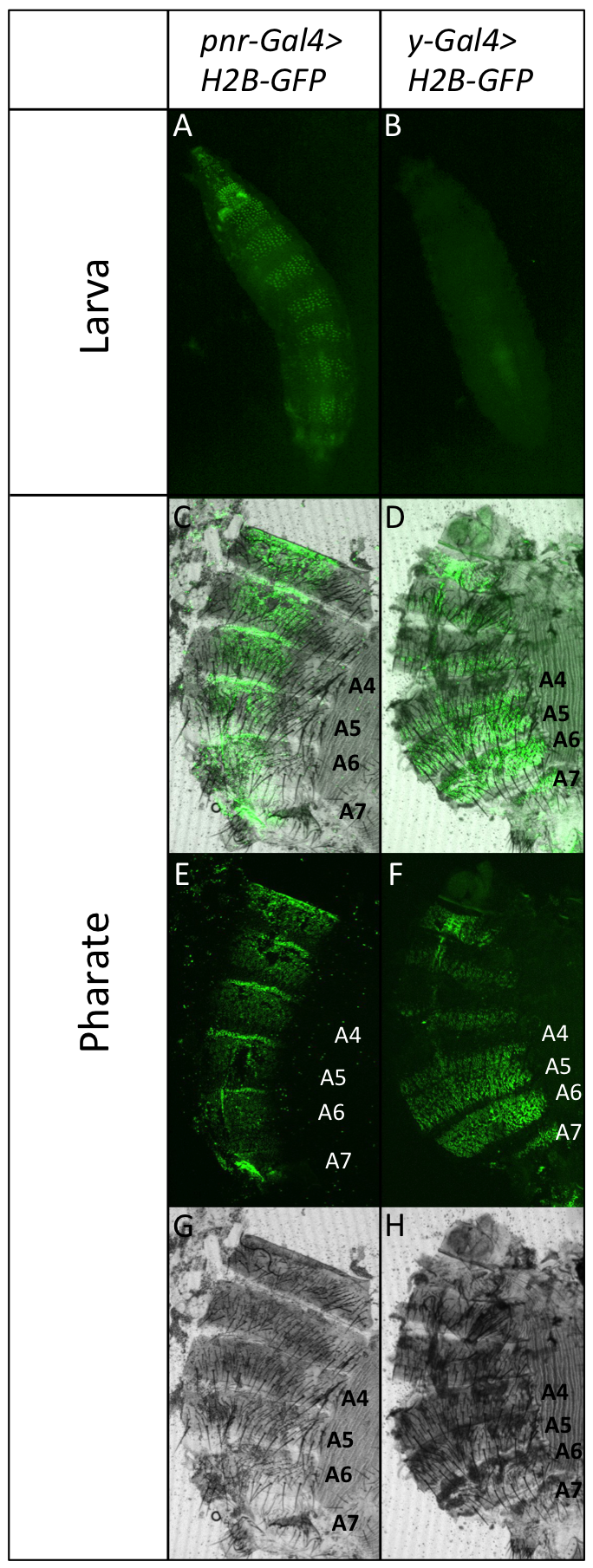
**Supplementary Figure 1 :**

Expression domains of *pnr-Gal4* and *y-Gal4* drivers during Drosophila development using *H2B-GFP* as a reporter gene. A, B, E, F: GFP expression in larvae and pharates; G, H: bright-field pictures to identify abdominal segments in pharates; C, D: merge of GFP expression and bright-field pictures.

*pnr-Gal4* is expressed along the dorsal midline in larvae (A) and pharates (C, E). Thus, the lateral regions in which Gal4 is not present can be used as internal controls.

*y-Gal4* is not expressed in larvae (B) and is expressed in abdominal segments of pharates (D, F), in the whole tergites in A5 to A7, and on the posterior border in A2 to A4.
