# Supplementary material for: The H3K79me3 methyl-transferase Grappa is involved in the establishment and thermal plasticity of abdominal pigmentation in Drosophila melanogaster females
Source: Sci Rep. 2024 Apr 25;14:9547. doi: 10.1038/s41598-024-60184-6 (PMC11045721; doi:10.1038/s41598-024-60184-6)
Supplement: Supplementary file 2 — Supplementary Information 2. [file 41598_2024_60184_MOESM2_ESM.docx]

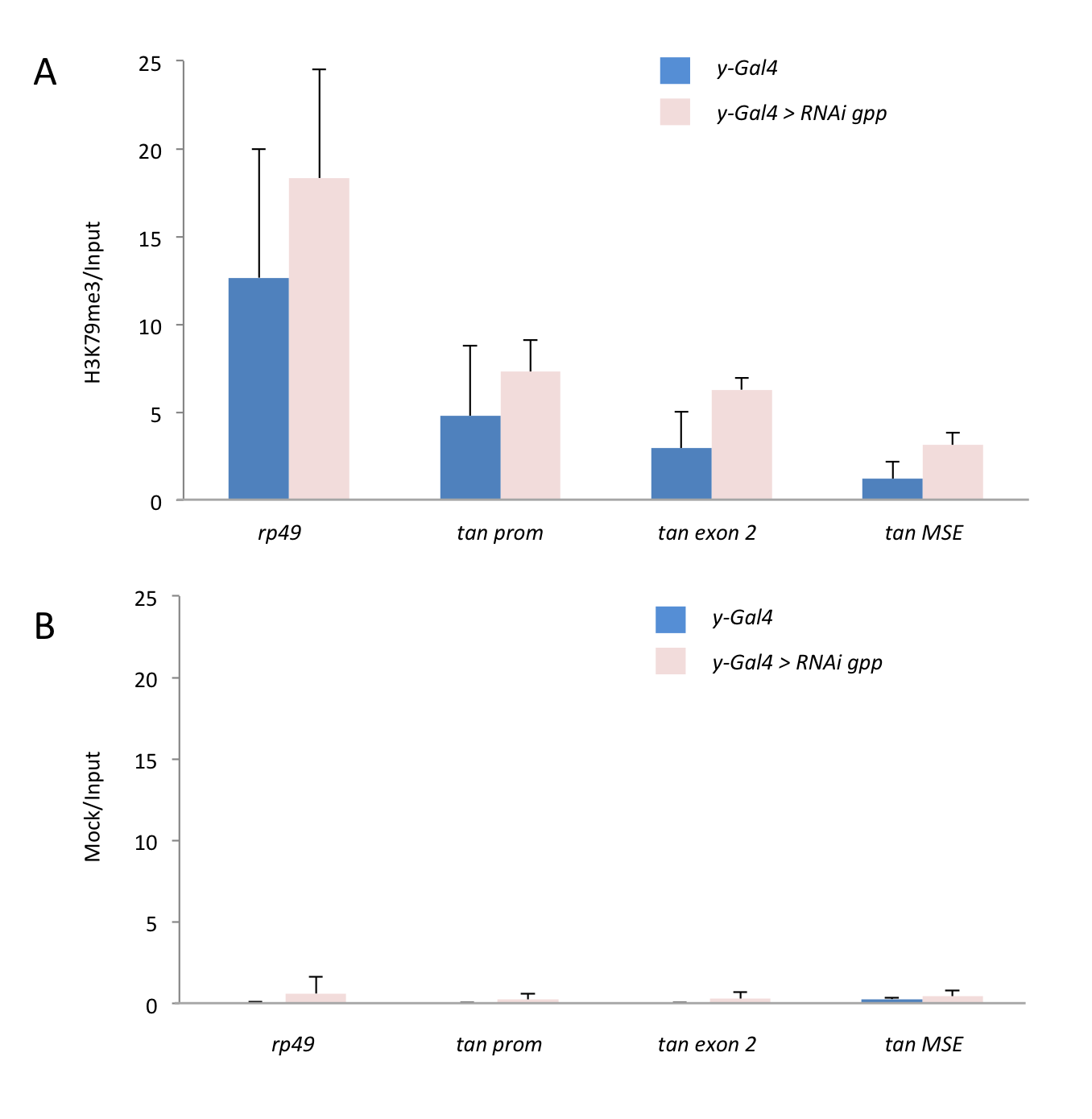


**Supplementary Figure 2:**  *gpp* down-regulation does not reduce the level of H3K79me3 on *tan* promoter, *tan* exon 2 and *tan* abdominal enhancer (*tan MSE*). Chromatin immunoprecipitation experiments (ChIP-qPCR) were performed on abdominal epidermis of young *y-Gal4*>*RNAi*-*gpp* and *y-Gal4/+* females grown at 25°C. H3K79me3 IP (A) and negative control IP (anti-Rabbit IgGs) (B) signals were normalized on total chromatin (input) signals. H3K79me level on *rp49* was used as a control. The graph represents the mean of three independent experiments, with error bars corresponding to standard deviations.
