# Supplementary material for: The H3K79me3 methyl-transferase Grappa is involved in the establishment and thermal plasticity of abdominal pigmentation in Drosophila melanogaster females
Source: Sci Rep. 2024 Apr 25;14:9547. doi: 10.1038/s41598-024-60184-6 (PMC11045721; doi:10.1038/s41598-024-60184-6)
Supplement: Supplementary file 3 — Supplementary Information 3. [file 41598_2024_60184_MOESM3_ESM.docx]

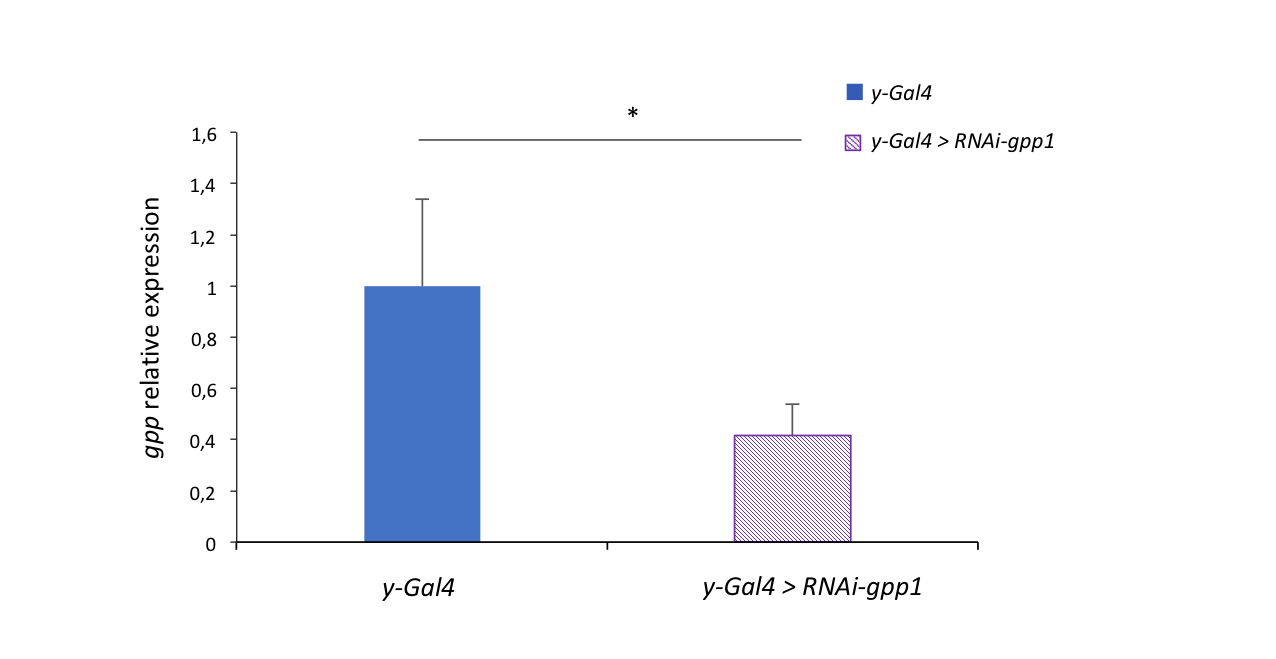


**Supplementary Figure 3:** Quantification by RT-qPCR of *gpp* expression to validate the efficiency of the *RNAi-ggp1* transgene.

RT-qPCR quantification of *gpp* expression in posterior abdomen epidermis (A5, A6 and A7 segments) of young females, *y-Gal4/+* (controls) or *y-Gal4 > RNAi-gpp1* (n = 3, t-test; *: p<0.05). Expression was normalized with the geometric mean of *RP49* and *Spt6* expression.
