# Supplementary material for: The H3K79me3 methyl-transferase Grappa is involved in the establishment and thermal plasticity of abdominal pigmentation in Drosophila melanogaster females
Source: Sci Rep. 2024 Apr 25;14:9547. doi: 10.1038/s41598-024-60184-6 (PMC11045721; doi:10.1038/s41598-024-60184-6)
Supplement: Supplementary file 4 — Supplementary Information 4. [file 41598_2024_60184_MOESM4_ESM.docx]

**Supplementary File 1 : ANOVAs on aligned rank transformed data for Figure 1**

***pnr-gal4* driver :**

A5

|  | Df | Df.res | F value | Pr(>F) |
| --- | --- | --- | --- | --- |
| Genotype | 2 | 87 | 14.958 | 2.61^E^-06 |

A6

|  | Df | Df.res | F value | Pr(>F) |
| --- | --- | --- | --- | --- |
| Genotype | 2 | 87 | 10.81 | 1.06^E^-4 |

A7

|  | Df | Df.res | F value | Pr(>F) |
| --- | --- | --- | --- | --- |
| Genotype | 2 | 87 | 1.9367 | 0.15035 |

***y-gal4 driver :***

A5

|  | Df | Df.res | F value | Pr(>F) |
| --- | --- | --- | --- | --- |
| Genotype | 2 | 87 | 69.278 | 2.22^E^-16 |

A6

|  | Df | Df.res | F value | Pr(>F) |
| --- | --- | --- | --- | --- |
| Genotype | 2 | 87 | 202.42 | 2.22^E^-16 |

A7

|  | Df | Df.res | F value | Pr(>F) |
| --- | --- | --- | --- | --- |
| Genotype | 2 | 87 | 61.287 | 2.22^E^-16 |
