# Supplementary material for: The H3K79me3 methyl-transferase Grappa is involved in the establishment and thermal plasticity of abdominal pigmentation in Drosophila melanogaster females
Source: Sci Rep. 2024 Apr 25;14:9547. doi: 10.1038/s41598-024-60184-6 (PMC11045721; doi:10.1038/s41598-024-60184-6)
Supplement: Supplementary file 6 — Supplementary Information 6. [file 41598_2024_60184_MOESM6_ESM.docx]

**Supplementary File 3 : Analyses of reaction norms for Figure 6**

A5 : ANOVA on aligned rank transformed data

|  | Df | Df.res | F value | Pr(>F) |
| --- | --- | --- | --- | --- |
| Genotype | 1 | 174 | 39.7152 | 2.3355^E^-09 |
| Temperature | 2 | 174 | 184.9464 | <2.22^E^-16 |
| GxT | 2 | 174 | 3.1598 | 0.044879 |

A6 : ANOVA

|  | Sum Sq | Df | F value | Pr(>F) |
| --- | --- | --- | --- | --- |
| (Intercept) | 5583838 | 1 | 1.1699^E^+5 | <2.2^E^-16 |
| Genotype | 32 | 1 | 6.7910^E^-01 | 0.411 |
| Temperature | 47740 | 2 | 5.0012^E^+02 | <2.2^E^-16 |
| GxT | 2038 | 2 | 2.12345^E^+01 | 5.125^E^-09 |
| Residuals | 8305 | 174 |  |  |

A7 : ANOVA

|  | Sum Sq | Df | F value | Pr(>F) |
| --- | --- | --- | --- | --- |
| (Intercept) | 4069330 | 1 | 39281.3502 | <2.2^E^-16 |
| Genotype | 24 | 1 | 0.2322 | 0.6305 |
| Temperature | 99497 | 2 | 480.2233 | <2.2^E^-16 |
| GxT | 3767 | 2 | 18.1821 | 6.749^E^-08 |
| Residuals | 18025 | 174 |  |  |
