# Supplementary material for: The H3K79me3 methyl-transferase Grappa is involved in the establishment and thermal plasticity of abdominal pigmentation in Drosophila melanogaster females
Source: Sci Rep. 2024 Apr 25;14:9547. doi: 10.1038/s41598-024-60184-6 (PMC11045721; doi:10.1038/s41598-024-60184-6)
Supplement: Supplementary file 11 — Supplementary Information 11. [file 41598_2024_60184_MOESM11_ESM.docx]

| Gene | Primer | Sequence (5'->3') | Experiment |
| --- | --- | --- | --- |
| *tan* | T-F | ACAGGGAAATGGTCCTGGCACTGA |  |
|  | T-R | TTTACTGCGTCTGGCGGGAGAA |  |
| *ebony* | E-F | CTGGAATGTGCTGGTGGAG |  |
|  | E-R | AGACCCTTGGGCAGGTAGTT |  |
| *TH* | TH-F | TGTGTATGTTTGGGTGGTGGGGGT |  |
|  | TH-R | AGTTGCGACGCCCGTTCTAAGT |  |
| *Ddc* | D-F | AGCATCTTGCCCAGCCAATCCA |  |
|  | D-R | TGAGTGGAGCGATTGCCTGCAT |  |
| *yellow* | Y-F | AGTGTGGTCGGCTGTGGGTTTT |  |
|  | Y-R | TGGATTTGTGTCCACGCCAGGT |  |
| *laccase2* | Lacs-F | CAATAGCCTTGGGTCTGGA |  |
|  | Lacs-R | TGGATAACGTGCAGAGGTCG | RT-qPCR |
| *black* | Black-F | TGCCAAGCCGCTGATTATCT |  |
|  | Black-R | TGTTTCTCCAGGTCGCTCAG |  |
| *bab1* | Bab1-F  Bab1-R | CAACTTGAATAAGCCCGCCG CCCTCAAACGAAGGACGGAG |  |
|  |  |  |  |
| *rp49* | RP49-F | CCGCTTCAAGGGACAGTATC |  |
|  | RP49-R | GACAATCTCCTTGCGCTTCT |  |
| *Spt6* | Act5C-F  Act5C-R | CGGAGGAGCTCTTCGATATG GACAGCTCTGGGAAGTCGTC |  |
| *grappa* | gpp-F | TGTTCCGTTCGCAGTACACA |  |
|  | gpp-R | CTGGCTGGCTCTGTTTTTCA |  |
| *tan-prom* | t-prom-F | CACGCTGTGTAGTTTGGGTT | ChIP-qPCR |
|  | t-prom-R | AGCTGATCTCTGGACTTGCT |  |
| *tan-exon 2* | t-ex2-F  t-ex2-R | GGTAGAGCGGCAAGTAGGTC  TCGCACATTTGGGTCCATGA |  |
| *tan-tMSE* | t_MSE-F  t_MSE-R | TTTGTTTCAACTCAATCCTAGCAG  TTTCAAGTGGTCTTGGTGCT |  |
| *rp49* | RP49-F | CCGCTTCAAGGGACAGTATC |  |
|  | RP49-R | GACAATCTCCTTGCGCTTCT |  |

**Supplementary table 3: list of primers used in the study**
